# Radiative Squeezing Flow of Second Grade Fluid with Convective Boundary Conditions

**DOI:** 10.1371/journal.pone.0152555

**Published:** 2016-04-20

**Authors:** T. Hayat, Sumaira Jabeen, Anum Shafiq, A. Alsaedi

**Affiliations:** 1 Department of Mathematics, Quaid-I-Azam University, 45320, Islamabad, 44000, Pakistan; 2 Nonlinear Analysis and Applied Mathematics (NAAM) Research Group, Department of Mathematics, Faculty of Science, King Abdulaziz University, Jeddah, 21589, Saudi Arabia; North China Electric Power University, CHINA

## Abstract

Influence of magnetohydrodynamic (MHD) flow between two parallel disks is considered. Heat transfer analysis is disclosed due to thermal radiation and convective boundary condition. Appropriate transformations are invoked to obtain the ordinary differential system. This system is solved using homotopic approach. Convergence of the obtained solution is discussed. Variations of embedded parameters into the governing problems are graphically discussed. Skin friction coefficient and Nusselt number are numerically computed and analyzed. It is noticed that temperature profile is increasing function of radiation parameter.

## Introduction

Squeezing flow has attracted significant attention of many researchers and scientists due to its paramount applications in various fields such as in bio-mechanics, food processing, mechanical, industrial and chemical engineering. This phenomenon is also observed in bearings, gears, rolling elements, machine tools, automotive engines, polymer processing, design of lubrication systems (including oil and grease systems) and injection and compression shaping etc. These type of flows are generated in many hydrodynamical machines and tools where vertical velocities or normal stresses are applying due to moving boundary. Stefan [1] was the first who studied the squeezing flows. He presented an adhoc asymptotic solution for flow of Newtonian fluid. Further Mahmood et al. [2] investigated the squeezed flow and heat transfer for viscous fluid towards a porous surface. Mustafa et al. [3] developed analytical solutions for the squeezing flow of nanofluid between the parallel disks. Qayyum et al. [4] examined the unsteady squeezed flow of Jeffery fluid between two parallel disks. Magnetohydrodynamic squeezing flow of second grade fluid between two parallel disks is studied by Hayat et al. [5]. Ganji et al. [6] investigated the magnetohydrodynamic (MHD) squeezed flow between two porous disks. Thermal radiation effects in time-dependent axisymmetric squeezing flow of Jeffery fluid between two parallel disks is analyzed by Hayat et al. [7]. Homotopy perturbation solution of MHD squeezing flow between two porous disks is studied by Domairry and Aziz [8].

Flow of electrically conducting fluid in presence of magnetic field is related to magnetohydrodynamics (MHD). The MHD systems are used in many applications such as power generators, accelerators, droplet filters, the design of heat exchangers, electrostatic filters, the cooling of reactors, pumps etc. Magnetic nanofluid is a fluid with unique characteristics of both liquid and magnetic field. Numerous applications involving magnetic nanofluids include drug delivery, contrast enhancement in magnetic resonance imaging and magnetic cell separation. Few representative studies on magnetohydrodynamics (MHD) nanofluid can be consulted through the investigations [9–18]. On the other hand the convective heat transfer has also mobilized substantial interest due to its significance in the industrial and environmental technologies including energy storage, gas turbines, nuclear plants, rocket propulsion, geothermal reservoirs and photovoltaic panels. The convective boundary condition has also attracted some interest and this usually is simulated via a Biot number in the wall thermal boundary condition. Recently Rashidi et al. [19] applied the one parameter continuous group method to investigate similarity solutions of magnetohydrodynamic (MHD) heat and mass transfer flow of viscous fluid over a flat surface with convective boundary condition. Analysis of heat and mass transfer in mixed convective flow towards a vertical flat surface with hydrodynamic slip and thermal convective boundary condition is examined by Rashidi et al. [20]. Bachok et al. [21] considered the stagnation point flow towards a shrinking or stretching surface with the bottom of surface heated through convection from a hot fluid. Magnetohydrodynamic (MHD) three-dimensional flow of an incompressible fluid induced by an exponentially stretching surface with convective boundary condition is investigated by Hayat et al. [22].

The radiation effects in the boundary layer flow is very important due to its application in physics, engineering and industrial fields such as glass production, furnace design, polymer processing, gas cooled nuclear reactors and also in space technology like aerodynamics rockets, missiles, propulsion system, power plants for inter planetary flights and space crafts operating at high temperatures. Hence thermal radiation effects cannot be ignored in such processes. Rosseland approximation is used to describe the radiative heat flux in the energy equation. Hayat et al. [23] examined the two-dimensional magnetohydrodynamic flow of thixotropic fluid towards a stretched surface with variable thermal conductivity and thermal radiation effects. Pal [24] examined the effects of Hall current in magnetohydrodynamic (MHD) flow and heat transfer characteristics of viscous fluid past an unsteady permeable radiative stretching surface. Bhattacharyya et al. [25] analyzed the rate of heat transfer in micropolar fluid flow towards a porous shrinking surface with thermal radiation. Heat transfer analysis in MHD flow of viscous fluid past an exponentially stretching sheet with suction/injection effects is conducted by Mukhopadhyay [26]. Hayat et al. [27] observed the mixed convection stagnation point flow of Maxwell fluid over a surface with convective boundary conditions and thermal radiation. Bhattacharyya [28] considered the MHD radiative flow of Casson fluid over a stretching sheet in the stagnation region. Sheikholeslami et al. [29] reported the effect of thermal radiation and heat transfer by considering two phase model in magnetohydrodynamic nanofluid flow.

The purpose of present paper is to analyze the analytic solution of squeezing flow of second grade fluid between two porous disks. Fluid is electrically conducting in presence of variable magnetic field. Heat transfer is carried out with thermal radiation and convective boundary condition. Series solutions are found by homotopy analysis method [30–40]. Velocity, temperature, skin friction coefficient and Nusselt number are analyzed for different emerging parameters. To check the validity of the solutions, we have presented the comparison of our results for limiting previously published papers [5,8]. An excellent agreement is found.

## Formulation

**Fig 1:**
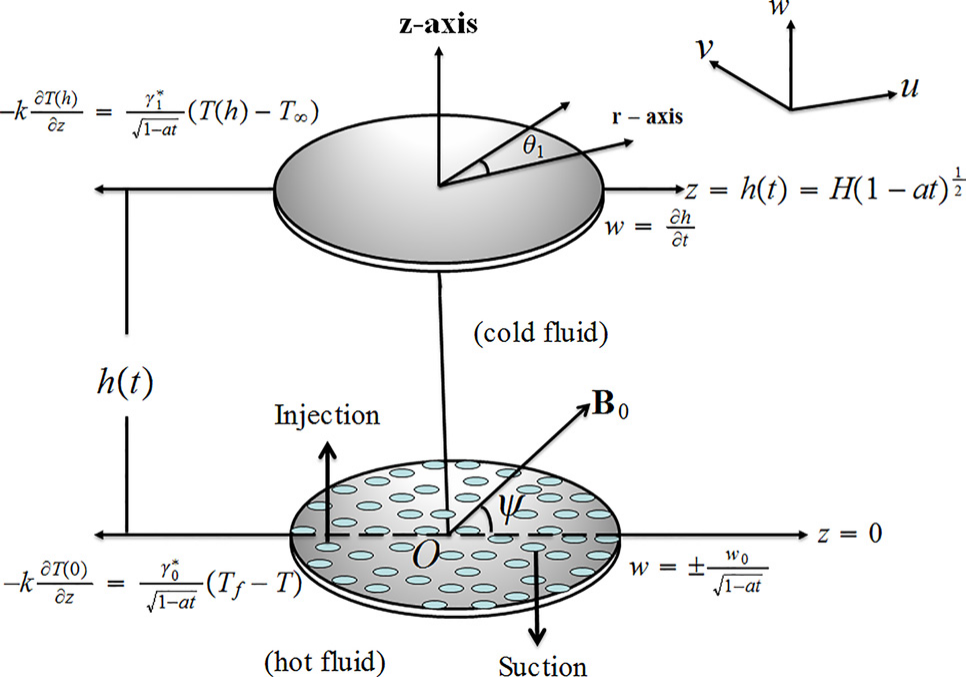
Geometry of problem.

We examine the incompressible axisymmetric squeezing flow of second-grade fluid between two infinite porous disks (see Fig 1). Heat transfer analysis is carried out by taking thermal radiation and convective boundary condition. The upper disk at $z=H{\left(1-at\right)}^{\frac{1}{2}}$ is moving with velocity $\frac{dz}{dt}=\frac{-1}{2}aH{\left(1-at\right)}^{\frac{-1}{2}}$ while lower porous disk at *z* = 0 is stationary. Mathematically $w=\frac{\partial h}{\partial t}$ represents the squeezing of the upper disk towards the lower disk. Variable magnetic field of inclined character via an angle *ψ* is applied. The law of conservation of mass, momentum and energy for the considered problem are

$$\frac{\partial u}{\partial r}+\frac{\partial w}{\partial z}+\frac{u}{r}=0,$$ (1)

$$\begin{array}{l} \rho \left(\frac{\partial u}{\partial t}+u\frac{\partial u}{\partial r}+w\frac{\partial u}{\partial z}\right)=-\frac{\partial p}{\partial r}+\mu \left(\frac{\partial^{2}u}{\partial z^{2}}+\frac{1}{r}\frac{\partial u}{\partial r}+\frac{\partial^{2}u}{\partial r^{2}}-\frac{u}{r^{2}}\right)+\alpha \{\frac{2}{r}\frac{\partial^{2}u}{\partial r\partial t}-\frac{2}{r^{2}}\frac{\partial u}{\partial t}\\ \;+2\frac{\partial^{3}u}{\partial r^{2}\partial t}+\frac{\partial^{3}w}{\partial r\partial z\partial t}+\frac{\partial^{3}u}{\partial z^{2}\partial t}+2\frac{u^{2}}{r^{3}}-2\frac{w}{r^{2}}\frac{\partial u}{\partial z}-\frac{1}{r}{\left(\frac{\partial u}{\partial z}\right)}^{2}\\ \;-\frac{\partial u}{\partial z}\frac{\partial^{2}w}{\partial z^{2}}+w\frac{\partial^{3}u}{\partial z^{3}}-2\frac{u}{r^{2}}\frac{\partial u}{\partial r}+\frac{\partial u}{\partial r}\frac{\partial^{2}u}{\partial z^{2}}+\frac{\partial w}{\partial r}\frac{\partial^{2}w}{\partial z^{2}}\\ \;+\frac{1}{r}{\left(\frac{\partial w}{\partial r}\right)}^{2}+2\frac{w}{r}\frac{\partial^{2}u}{\partial r\partial z}+\frac{\partial w}{\partial r}\frac{\partial^{2}u}{\partial r\partial z}+2\frac{\partial w}{\partial z}\frac{\partial^{2}w}{\partial r\partial z}\\ \;-\frac{\partial u}{\partial r}\frac{\partial^{2}w}{\partial r\partial z}+u\frac{\partial^{3}u}{\partial r\partial z^{2}}+w\frac{\partial^{3}w}{\partial r\partial z^{2}}+2\frac{u}{r}\frac{\partial^{2}u}{\partial r^{2}}+2\frac{\partial u}{\partial r}\frac{\partial^{2}u}{\partial r^{2}}\\ \;+\frac{\partial u}{\partial z}\frac{\partial^{2}w}{\partial r^{2}}+2\frac{\partial w}{\partial r}\frac{\partial^{2}w}{\partial r^{2}}+2w\frac{\partial^{3}u}{\partial r^{2}\partial z}+u\frac{\partial^{3}w}{\partial r^{2}\partial z}+2u\frac{\partial^{3}u}{\partial r^{3}}\}\\ \;-\frac{\sigma B_{0}^{2}}{\left(1-at\right)}\sin^{2}\psi u, \end{array}$$ (2)

$$\begin{array}{l} \rho \left(\frac{\partial w}{\partial t}+u\frac{\partial w}{\partial r}+w\frac{\partial w}{\partial z}\right)=-\frac{\partial p}{\partial z}+\mu \left(\frac{1}{r}\frac{\partial w}{\partial r}+\frac{\partial^{2}w}{\partial r^{2}}+\frac{\partial^{2}w}{\partial z^{2}}\right)+\alpha \{\frac{1}{r}\frac{\partial^{2}u}{\partial z\partial t}+\frac{1}{r}\frac{\partial^{2}w}{\partial r\partial t}\\ \;+\frac{\partial^{3}w}{\partial r^{2}\partial t}+\frac{\partial^{3}u}{\partial r\partial z\partial t}+2\frac{\partial^{3}w}{\partial z^{2}\partial t}-\frac{1}{r}\frac{\partial u}{\partial z}\frac{\partial w}{\partial z}+\frac{w}{r}\frac{\partial^{2}u}{\partial z^{2}}\\ \;+2\frac{\partial u}{\partial z}\frac{\partial^{2}u}{\partial z^{2}}+2\frac{\partial w}{\partial z}\frac{\partial^{2}w}{\partial z^{2}}+2w\frac{\partial^{3}w}{\partial z^{3}}+\frac{1}{r}\frac{\partial u}{\partial z}\frac{\partial u}{\partial r}+\frac{1}{r}\frac{\partial w}{\partial z}\frac{\partial w}{\partial r}\\ \;+\frac{\partial w}{\partial r}\frac{\partial^{2}u}{\partial z^{2}}-\frac{1}{r}\frac{\partial u}{\partial r}\frac{\partial w}{\partial r}+\frac{u}{r}\frac{\partial^{2}u}{\partial z\partial r}-\frac{\partial w}{\partial z}\frac{\partial^{2}u}{\partial r\partial z}+2\frac{\partial u}{\partial r}\frac{\partial^{2}u}{\partial r\partial z}\\ \;+\frac{w}{r}\frac{\partial^{2}w}{\partial r\partial z}+\frac{\partial u}{\partial z}\frac{\partial^{2}w}{\partial r\partial z}+w\frac{\partial^{3}u}{\partial r\partial z^{2}}+2u\frac{\partial^{3}w}{\partial r\partial z^{2}}+\frac{\partial u}{\partial z}\frac{\partial^{2}u}{\partial r^{2}}\\ \;-\frac{\partial w}{\partial r}\frac{\partial^{2}u}{\partial r^{2}}+\frac{u}{r}\frac{\partial^{2}w}{\partial r^{2}}+\frac{\partial w}{\partial z}\frac{\partial^{2}w}{\partial r^{2}}+u\frac{\partial^{3}u}{\partial r^{2}\partial z}+w\frac{\partial^{3}w}{\partial r^{2}\partial z}\\ \;+u\frac{\partial^{3}w}{\partial r^{3}}\}-\frac{\sigma B_{0}^{2}}{\left(1-at\right)}\cos^{2}\psi w, \end{array}$$ (3)

$$\rho c_{p}\left(\frac{\partial T}{\partial t}+u\frac{\partial T}{\partial r}+w\frac{\partial T}{\partial z}\right)=k\left(\frac{\partial^{2}T}{\partial r^{2}}+\frac{\partial^{2}T}{\partial z^{2}}+\frac{1}{r}\frac{\partial T}{\partial r}\right)+\frac{16\sigma^{*}T_{\infty }^{3}}{3k^{*}}\left(\frac{\partial^{2}T}{\partial r^{2}}+\frac{\partial^{2}T}{\partial z^{2}}+\frac{1}{r}\frac{\partial T}{\partial r}\right),$$ (4)

subject to the boundary conditions

$$u=0,\;w=\frac{\partial h}{\partial t},\;-k\frac{\partial T(h)}{\partial z}=\frac{\gamma_{1}^{*}}{\sqrt{1-at}}(T(h)-T_{\infty })\;\text{at}\;z=h(t),$$ (5)

$$u=0,\;w=\pm \frac{w_{0}}{\sqrt{1-at}},\;-k\frac{\partial T(0)}{\partial z}=\frac{\gamma_{0}^{*}}{\sqrt{1-at}}(T_{f}-T)\;\text{at}\;z=0.$$ (6)

In the above expressions *u* and *w* denote the velocity components in the *r* and *z* directions respectively, *ρ* the fluid density, *σ* the electrical conductivity, *c*_*p*_ the specific heat, *μ* the dynamic viscosity, $\gamma_{0}^{*}$ the heat transfer coefficient at the lower plate, $\gamma_{1}^{*}$ the rate of heat transfer coefficient of fluid at upper plate, *k* the thermal conductivity far away from the disk, σ* the Stefan-Boltzmann constant and k* the mean absorption coefficient, *w*_0_ corresponds to the case for suction while −*w*_0_ leads to the case for blowing. It should be noted that the governing equations are reduced to viscous fluid when *α* = 0.

We consider

$$\begin{array}{l} u=\frac{ar}{2(1-at)}f^{\prime }(\eta ),\;w=-\frac{aH}{\sqrt{1-at}}f(\eta ),\\ B=\frac{B_{0}}{\sqrt{1-at}},\;\eta =\frac{z}{H\sqrt{1-at}},\\ \theta =\frac{T-T_{\infty }}{T_{f}-T_{\infty }}, \end{array}$$ (7)

where *T*_*f*_ is the temperature of the hot fluid at the lower permeable disk, *T*_∞_ the ambient temperature above the upper disk, *T* the fluid temperature such that *T*_*f*_ > *T* and *T*_∞_ < *T*. The Eq (1) is identically satisfied and Eqs (2–6) take the forms

$$f^{iv}-S(\eta f^{\prime \prime \prime }+3f^{\prime \prime }-2ff^{\prime \prime \prime })+\frac{\alpha_{1}}{2}(5f^{iv}+\eta f^{v}-2ff^{v})-\sin^{2}\psi M^{2}f^{\prime \prime }=0,$$ (8)

$$\left(1+\frac{4}{3}R\right)\theta^{\prime \prime }(\eta )-S\mathrm{Pr}\left[\eta \theta^{\prime }(\eta )-2\theta^{\prime }(\eta )f(\eta )\right]=0,$$ (9)

$$\begin{array}{l} f^{\prime }\left(\eta \right)=0,\;f(\eta )=A\;\theta^{\prime }(\eta )=\gamma_{1}(\theta (0)-1)\;\text{at}\;\eta =0,\\ f^{\prime }\left(\eta \right)=0,\;f(\eta )=\frac{1}{2}\;\theta^{\prime }(\eta )=-\gamma_{2}(\theta (1))\;\text{at}\;\eta =1, \end{array}$$ (10)

in which

$$\begin{array}{l} M=\sqrt{\frac{\sigma B_{0}^{2}H^{2}}{\mu }},\;S=\frac{aH^{2}}{2\upsilon },\;\alpha_{1}=\frac{\alpha a}{\mu (1-at)},\\ \;R=\frac{4\sigma^{*}T_{\infty }^{3}}{kk^{*}},\;\mathrm{Pr}=\frac{\mu c_{p}}{k},\;A=\frac{\pm w_{0}}{aH},\\ \gamma_{1}=\frac{\gamma_{0}^{*}H}{k},\;\gamma_{2}=\frac{\gamma_{1}^{*}H}{k} \end{array}$$ (11)

where *M* denotes the magnetic parameter, *S* the squeezing parameter, *α*_1_ the dimensionless second grade fluid parameter, *R* represents radiation parameter and *A* represents suction/blowing parameter.

The skin friction coefficient and the local Nusselt number are

$$C_{fr}=\frac{{\tau_{rz}|}_{z=0,h(t)}}{\rho {\left(\frac{-aH}{2{\left(1-at\right)}^{\frac{1}{2}}}\right)}^{2}},$$ (12)

where

$$\begin{array}{l} \tau_{rz}=\mu \left(\frac{\partial u}{\partial z}+\frac{\partial w}{\partial r}\right)+\alpha (\frac{\partial^{2}u}{\partial z\partial t}+u\frac{\partial^{2}u}{\partial r\partial z}+w\frac{\partial^{2}u}{\partial z^{2}}+\frac{\partial^{2}w}{\partial r\partial t}+u\frac{\partial^{2}w}{\partial r^{2}}+w\frac{\partial^{2}w}{\partial r\partial z}\\ \;-\frac{\partial w}{\partial z}\frac{\partial u}{\partial z}+\frac{\partial w}{\partial z}\frac{\partial w}{\partial r}+\frac{\partial u}{\partial z}\frac{\partial u}{\partial r}-\frac{\partial w}{\partial r}\frac{\partial u}{\partial r}), \end{array}$$ (13)

and

$$Nu_{r}=\frac{Hq_{w}}{k\left(T_{f}-T_{\infty }\right)}=-\frac{H{\left(k+\frac{16\sigma^{*}T_{\infty }^{3}}{3k^{*}}\right)\frac{\partial T}{\partial z}|}_{z=0,h(t)}}{k\left(T_{f}-T_{\infty }\right)}.$$ (14)

In dimensionless form we obtain

$$\begin{array}{l} C_{fr1}\frac{H^{2}}{r^{2}}Re_{r}=\left(1+\frac{3}{2}\alpha_{1}\right)f^{\prime \prime }(1),\;C_{fr0}\frac{H^{2}}{r^{2}}Re_{r}=\left(1+\frac{3}{2}\alpha_{1}\right)f^{\prime \prime }(0)-\alpha_{1}Af^{\prime \prime \prime }(0),\\ \;\sqrt{1-at}Nu_{r1}=-\theta^{\prime }(1)\left(1+\frac{4}{3}R\right),\;\sqrt{1-at}Nu_{r0}=-\theta^{\prime }(0)\left(1+\frac{4}{3}R\right), \end{array}$$ (15)

in which $Re_{r}=\frac{arH{\left(1-at\right)}^{\frac{1}{2}}}{2\upsilon }$ is the local Reynold number, *Nu*_*r*0_ denotes the heat transfer rate at lower disk while *Nu*_*r*1_ is the heat transfer rate at upper disk. Similarly *C*_*fr*1_ and *C*_*fr*0_ represent the skin friction coefficient at upper and lower disks respectively.

## Homotopic Solutions

In order to find the homotopy analysis solutions we choose the base functions:

$$\left\{\eta^{n};n\geq 0\right\},$$ (16)

and express

$$f\left(\eta \right)=\overset{\infty }{\underset{n=0}{\sum }}a_{n}\eta^{n},$$ (17)

$$\theta \left(\eta \right)=\overset{\infty }{\underset{n=0}{\sum }}b_{n}\eta^{n},$$ (18)

where *a*_*n*_ and *b*_*n*_ are the coefficients to be determined. The initial guesses (*f*_0_,*θ*_0_) and auxiliary linear operators (**L**_*f*_,**L**_*θ*_) are

$$f_{0}(\eta )=(-1+2A)\eta^{3}+3\left(\frac{1}{2}-A\right)\eta^{2}+A,\;\theta_{0}(\eta )=\frac{\gamma_{1}(1+\gamma_{2}-\eta \gamma_{2})}{\gamma_{1}+\gamma_{2}+\gamma_{1}\gamma_{2}},$$ (19)

$$L_{f}\left(\eta \right)=\frac{d^{4}f}{d\eta^{4}},\;L_{\theta }\left(\eta \right)=\frac{d^{2}\theta }{d\eta^{2}},$$ (20)

$$L_{f}\;\left[C_{1}+C_{2}\eta^{3}+C_{3}\eta^{2}+C_{4}\eta \right]=0,$$ (21)

$$L_{\theta }\left[C_{5}+C_{6}\eta \right]=0,$$ (22)

with *C*_*i*_ (*i* = 1–6) denote the arbitrary constants.

### Zeroth-order problem

The zeroth order deformation problems are given below

$$(1-p)L_{f}[\hat{f}(\eta ,p)-f_{0}(\eta )]=p\hbar_{f}N_{f}\left[\hat{f}(\eta ,p)\right],$$ (23)

$${\frac{\partial \hat{f}(\eta ;p)}{\partial \eta }|}_{\eta =0}=0,\;{\frac{\partial \hat{f}(\eta ;p)}{\partial \eta }|}_{\eta =1}=0,\;{\hat{f}(\eta ;p)|}_{\eta =0}=A,\;{\hat{f}(\eta ;p)|}_{\eta =1}=\frac{1}{2},$$ (24)

$$(1-p)L_{\theta }[\hat{\theta }(\eta ,p)-\theta_{0}(\eta )]=p\hbar_{\theta }N_{\theta }\left[\hat{\theta }(\eta ,p),\hat{f}(\eta ,p)\right],$$ (25)

$${\frac{\partial \hat{\theta }(\eta ;p)}{\partial \eta }|}_{\eta =0}=\gamma_{1}(\theta (0)-1),\;{\frac{\partial \hat{\theta }(\eta ;p)}{\partial \eta }|}_{\eta =1}=-\gamma_{2}(\theta (1)),$$ (26)

$$\begin{array}{l} N_{f}\left[\hat{f}(\eta ;p)\right]=\frac{\partial^{4}\hat{f}(\eta ,p)}{\partial \eta^{4}}-S\left(3\frac{\partial^{2}\hat{f}(\eta ,p)}{\partial \eta^{2}}+\eta \frac{\partial^{3}\hat{f}(\eta ,p)}{\partial \eta^{3}}-2\hat{f}(\eta ,p)\frac{\partial^{3}\hat{f}(\eta ,p)}{\partial \eta^{3}}\right)\\ \;+\frac{\alpha_{1}}{2}\left(5\frac{\partial^{4}\hat{f}(\eta ,p)}{\partial \eta^{4}}+\eta \frac{\partial^{5}\hat{f}(\eta ,p)}{\partial \eta^{5}}-2\hat{f}(\eta ,p)\frac{\partial^{5}\hat{f}(\eta ,p)}{\partial \eta^{5}}\right)\\ \;-\sin^{2}\psi M^{2}\frac{\partial^{2}\hat{f}(\eta ,p)}{\partial \eta^{2}}, \end{array}$$ (27)

$$N_{\theta }\left[\hat{f}(\eta ;p),\hat{\theta }(\eta ;p)\right]=\left(1+\frac{4}{3}R\right)\frac{\partial^{2}\hat{\theta }(\eta ,p)}{\partial \eta^{2}}-\mathrm{Pr}S\left(\eta \frac{\partial \hat{\theta }(\eta ,p)}{\partial \eta }-2\frac{\partial \hat{\theta }(\eta ,p)}{\partial \eta }\hat{f}(\eta ,p)\right)$$ (28)

where, *p* ∈ [0,1] represents embedding parameter while ℏ_*f*_ and ℏ_*θ*_ denote non-zero auxiliary parameters.

### mth-order deformation problems

The *m*th-order deformation problems are

$$L_{f}\left[{\hat{f}}_{m}\left(\eta \right)-\chi_{m}{\hat{f}}_{m-1}\left(\eta \right)\right]=\hbar_{f}R_{m}^{f}\left(\eta \right),$$ (29)

$${\frac{\partial {\hat{f}}_{m}(\eta ,p)}{\partial \eta }|}_{\eta =0}=0,\;{\frac{\partial {\hat{f}}_{m}(\eta ,p)}{\partial \eta }|}_{\eta =1}=0,\;{{\hat{f}}_{m}(\eta ;p)|}_{\eta =0}=0,\;{{\hat{f}}_{m}(\eta ;p)|}_{\eta =1}=0,$$ (30)

$$L_{\theta }\left[{\hat{\theta }}_{m}\left(\eta \right)-\chi_{m}{\hat{\theta }}_{m-1}\left(\eta \right)\right]=\hbar_{\theta }R_{m}^{\theta }\left(\eta \right),$$ (31)

$${\frac{\partial {\hat{\theta }}_{m}(\eta ;p)}{\partial \eta }|}_{\eta =0}=0,\;{\frac{\partial {\hat{\theta }}_{m}(\eta ;p)}{\partial \eta }|}_{\eta =1}=0,$$ (32)

$$\begin{array}{l} R_{m}^{f}\left(\eta \right)=f_{m-1}^{iv}\left(\eta \right)-S\left(3f_{m-1}^{\prime \prime }(\eta )+\eta f_{m-1}^{\prime \prime \prime }(\eta )\right)+\frac{\alpha_{1}}{2}(5f_{m-1}^{iv}(\eta )\\ \;+\eta f_{m-1}^{v}(\eta ))+\sum_{k=0}^{m-1}\left[2S\left(f_{m-1-k}f_{k}^{\prime \prime \prime }\right)-\alpha_{1}f_{m-1-k}f_{k}^{v}\right]\\ \;-\sin^{2}\psi M^{2}f_{m-1}^{\prime \prime }(\eta ), \end{array}$$ (33)

$$R_{m}^{\theta }\left(\eta \right)=\left(1+\frac{4}{3}R\right)\theta^{\prime \prime }(\eta )-\mathrm{Pr}S\left(\eta \theta_{m-1}^{\prime }-2\overset{m-1}{\underset{k=0}{\sum }}f_{m-1-k}\theta_{k}^{\prime }\right),$$ (34)

$$\chi_{m}=\{\begin{array}{c} 0,\;m\leq 1\\ 1,\;m>1 \end{array}.$$ (35)

For *p* = 0 and *p* = 1 one has

$$\hat{f}(\eta ,0)=f_{0}(\eta ),\;\hat{f}(\eta ,1)=f(\eta ),$$ (36)

$$\hat{\theta }(\eta ,0)=\theta_{0}(\eta ),\;\hat{\theta }(\eta ,1)=\theta (\eta ).$$ (37)

When *p* varies from 0 to 1 then $\hat{f}(\eta ;p)$ and $\hat{\theta }(\eta ;p)$ start from the initial solutions *f*_0_(*η*) and *θ*_0_(*η*) and reach to the final solutions *f*(*η*) and *θ*(*η*) respectively. The values of auxiliary parameters is selected in such a manner that the series solutions converge. The general solutions (*f*_*m*_,*θ*_*m*_) via special solutions $\left(f_{m}^{*},\theta_{m}^{*}\right)$ are

$$f_{m}(\eta )=f_{m}^{*}(\eta )+C_{1}+C_{2}\eta^{3}+C_{3}\eta^{2}+C_{4}\eta ,$$ (38)

$$\theta_{m}(\eta )=\theta_{m}^{*}(\eta )+C_{5}+C_{6}\eta ,$$ (39)

where the *C*_*i*_ (*i* = 1–6) are the involved constants.

## Analysis of Results

### Convergence of solutions

**Fig 2:**
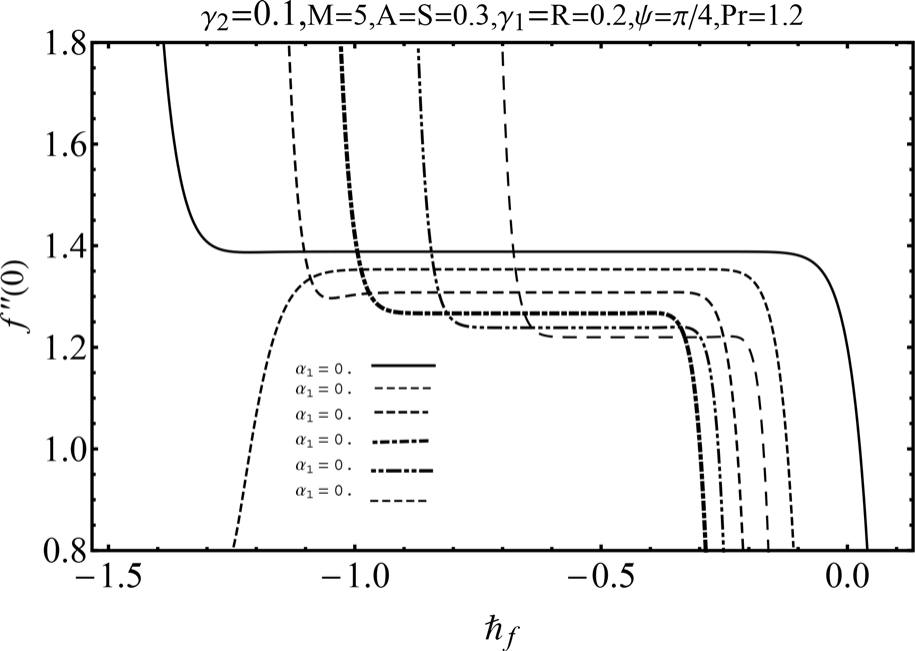
ℏ− curve for f″(0) via various values of *α*_1_.

**Fig 3:**
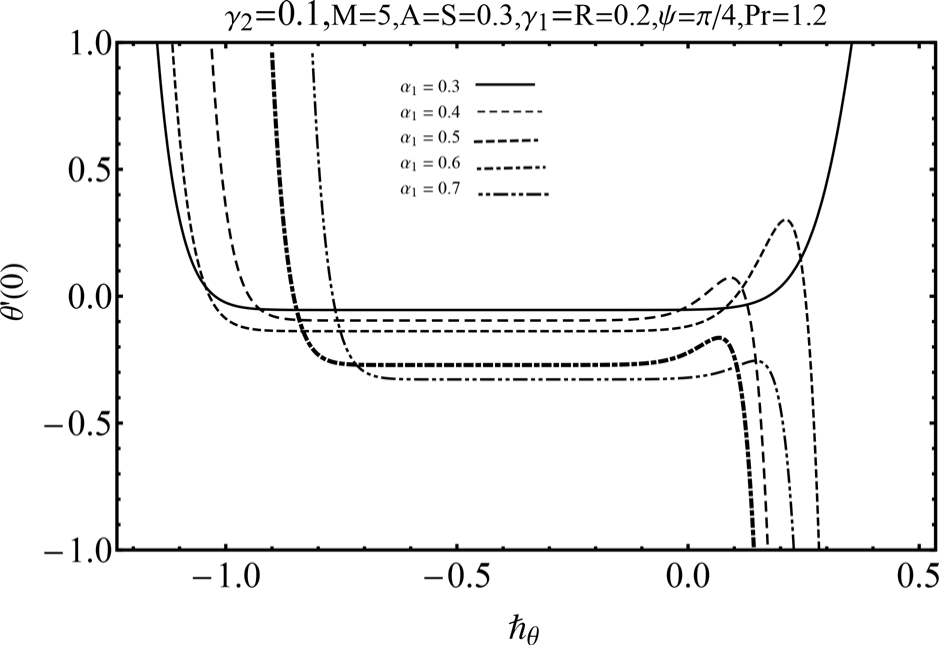
ℏ− curve for *θ*′(0) via various values of *α*_1_.

It is quite clear that derived series solutions contain the auxiliary parameters ℏ_*f*_ and ℏ_*θ*_ which are very important in controlling and adjusting the convergence. To obtain the admissible values of auxiliary parameters, the ℏ− curves are sketched at 19^*th*^-order of approximation in Figs 2 and 3. These curves are sketched for different values of second grade fluid parameter *α*_1_ for both velocity and temperature. These Figs show that meaningful values of ℏ_*f*_ and ℏ_*θ*_ are −1.3 ≤ ℏ_*f*_ < −0.2 (for *α*_1_ = 0.1) and −0.9 ≤ ℏ_*θ*_ < −0.2 (for *α*_1_ = 0.3). Also Table 1 depicts that the series solutions are convergent up to six decimal places at 8^*th*^ order of approximation for momentum and 6^*th*^ order of approximation for temperature.

**Table 1:** Convergence of HAM solutions for different order of approximations when *M* = 5, *S* = *A* = 0.3, *α*_1_ = *γ*_2_ = 0.1, *R* = *γ*_1_ = 0.2, Pr = 1.2 and *ψ* = *π*/4.

Order of approximations	*f*″(0)	−*θ*′(0)
1	1.460023	0.066542
4	1.387792	0.066097
6	1.388400	0.066093
8	1.388285	0.066093
12	1.388285	0.066093
20	1.388285	0.066093
30	1.388285	0.066093
50	1.388285	0.066093

Residual errors are calculated for momentum and energy equations by using expressions

$$\Delta_{m}^{f}=\int_{0}^{1}{\left[R_{m}^{f}\left(\eta ,\hbar_{f}\right)\right]}^{2}d\eta ,$$ (40)

$$\Delta_{m}^{\theta }=\int_{0}^{1}{\left[R_{m}^{\theta }\left(\eta ,\hbar_{\theta }\right)\right]}^{2}d\eta .$$ (41)

**Fig 4:**
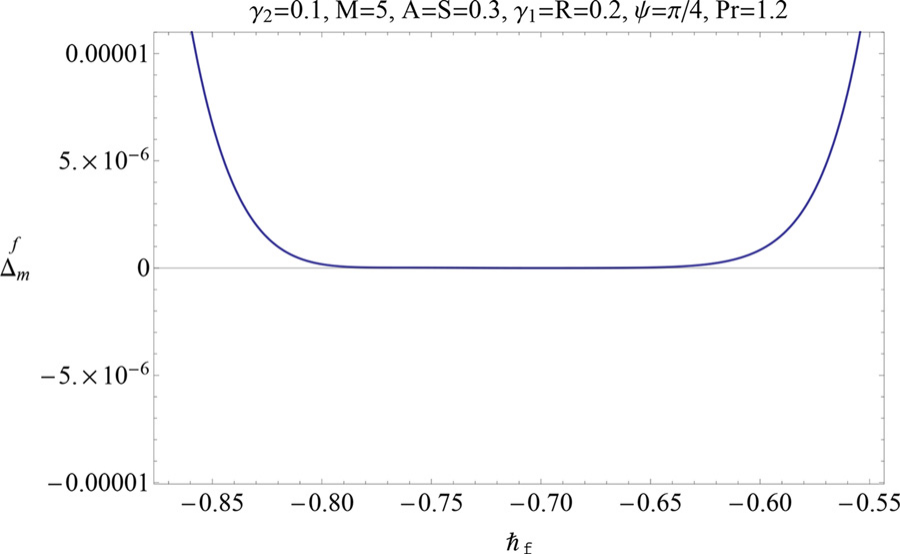
Residual error for ℏ_*f*_.

**Fig 5:**
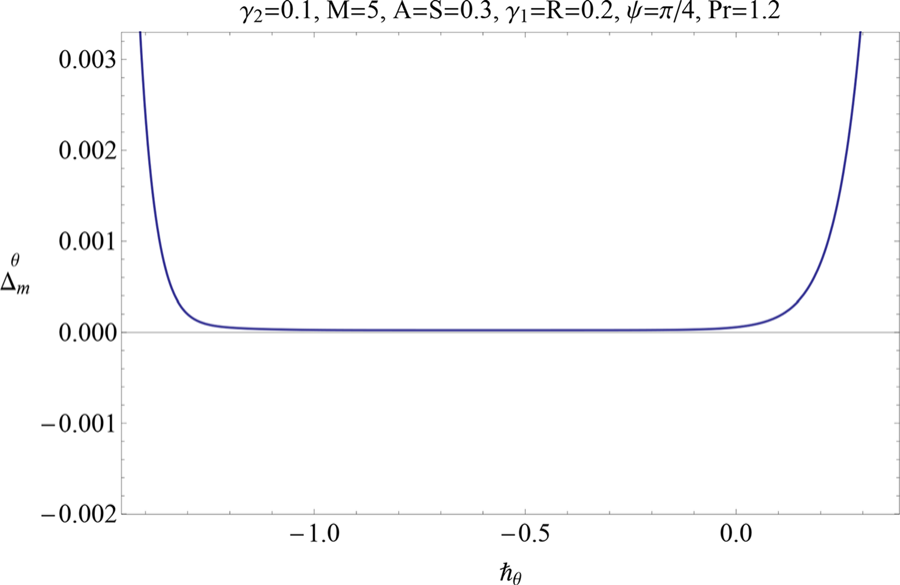
Residual error for ℏ_*θ*_.

Figs 4 and 5 display the ℏ− curves for residual error of *ƒ* and *θ* in order to get the admissible range for ℏ. It is observed that correct result up to 6th decimal places is obtained by choosing the value of ℏ from this range. Further the series solutions converge in the whole region of *η*(0 < *η* < ∞) when ℏ_*f*_ = −0.9 = ℏ_*θ*_.

## Discussion

In this subsection, we studied the influence of diverse parameters on the velocity, temperature, skin friction coefficient and local Nusselt number.

### Velocity profile

**Fig 6:**
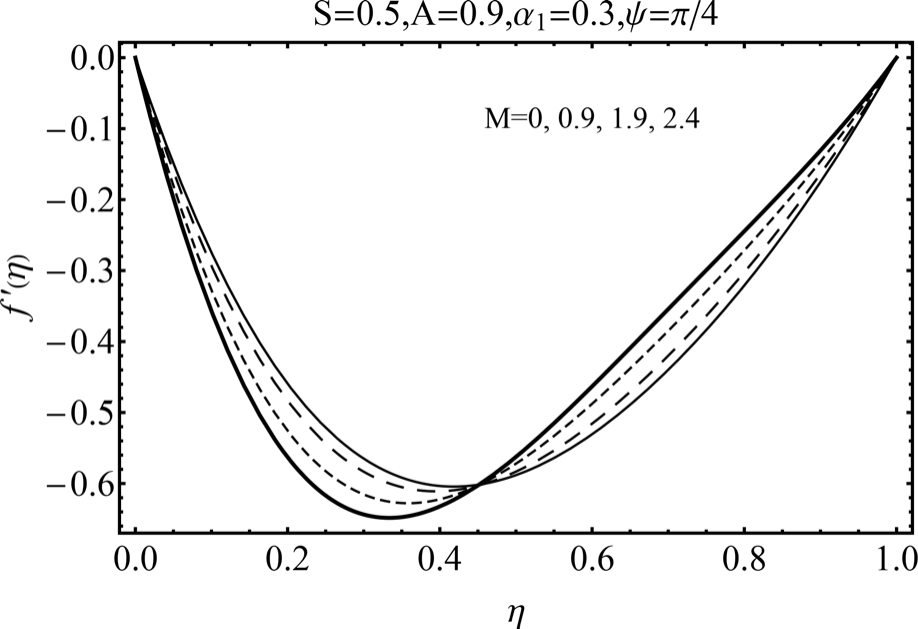
Influence of *M* on\*f*′(*η*) for suction.

**Fig 7:**
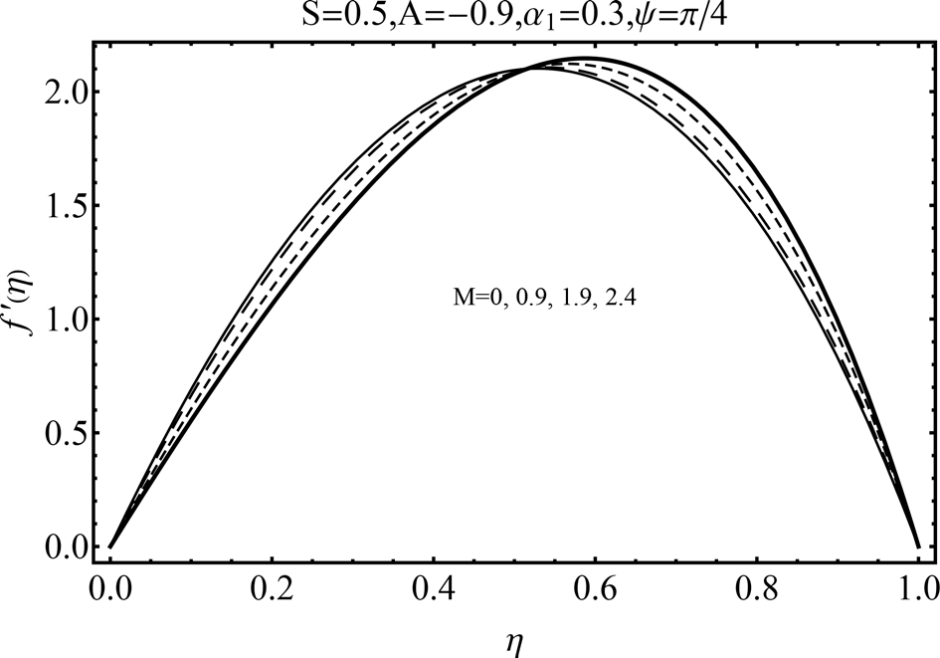
Influence of *M* on *f*′(*η*) for blowing.

**Fig 8:**
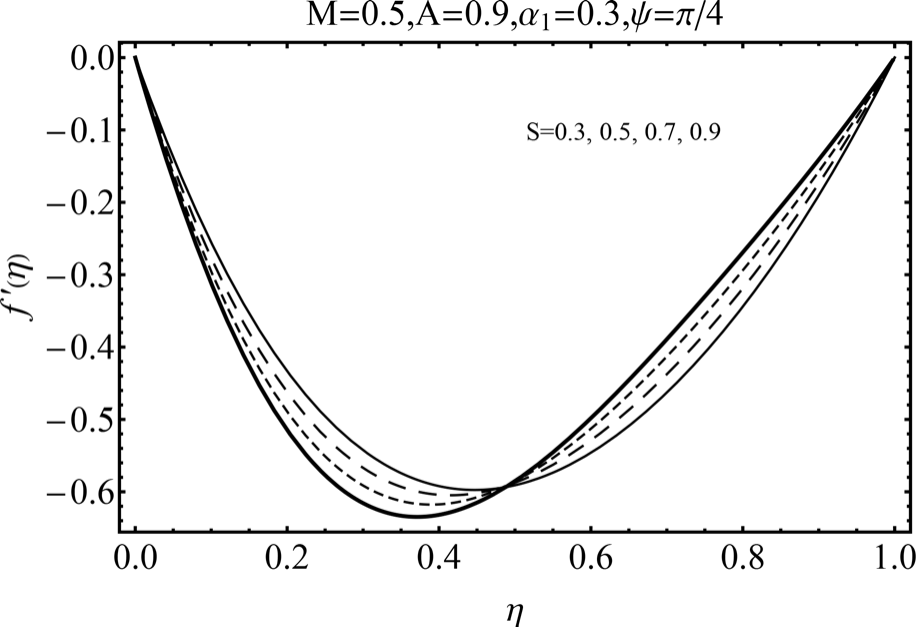
Influence of *S* on *f*′(*η*) for suction.

**Fig 9:**
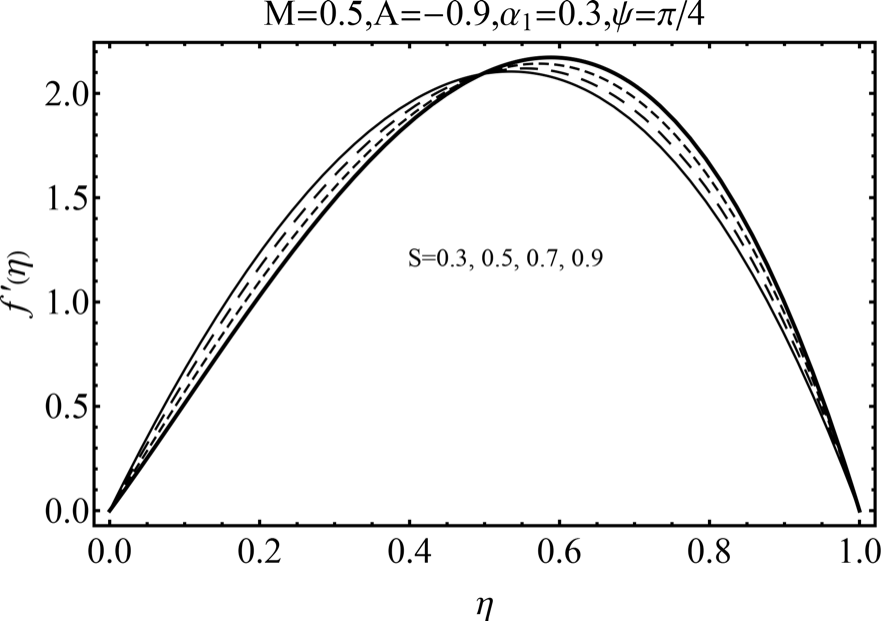
Influence of *S* on *f*′(*η*) for blowing.

**Fig 10:**
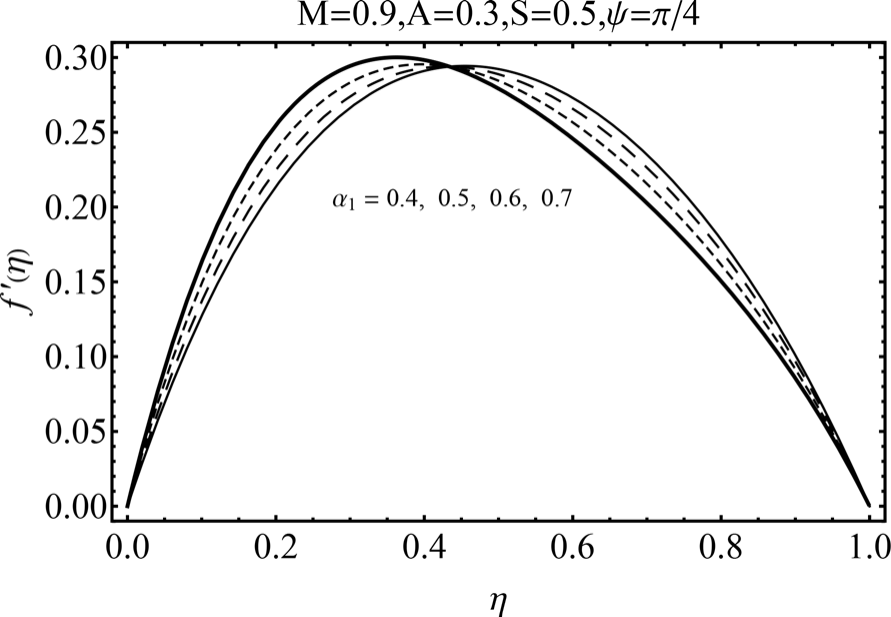
Influence of *α*_1_ on *f*′(*η*).

**Fig 11:**
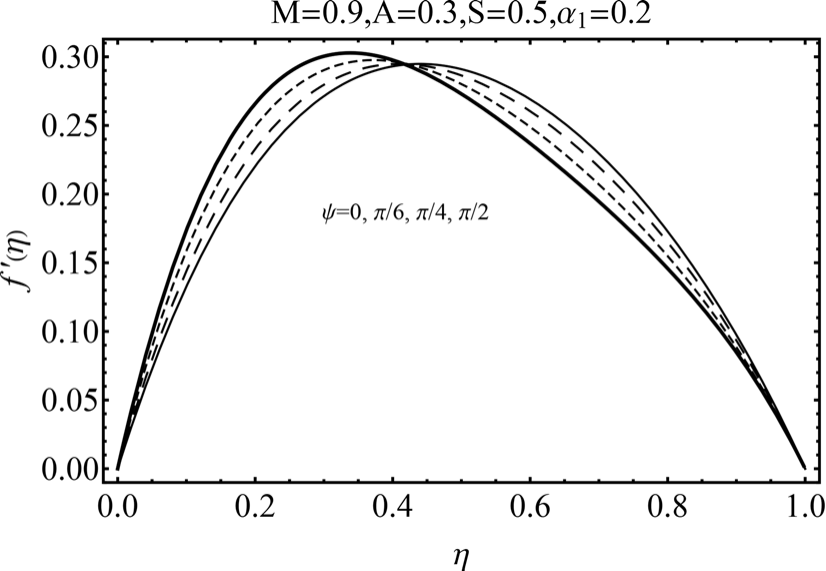
Influence of *ψ* on *f*′(*η*).

Effect of magnetic parameter (*M*) on the velocity distribution *f*′(*η*) in cases of suction and blowing have been displayed through Figs 6 and 7. It is observed that the magnitude of the radial velocity *f*′(*η*) shows dual behavior with the increase in the magnetic parameter *M*. For higher magnetic parameter *M*, the velocity profile decreases near the porous walls where the suction effects are dominant when *η* ≤ 0.42. However for *η* > 0.42 the velocity profile increases. In fact the higher magnetic field slows down the fluid particles (because of the resistive force known as Lorentz force) and consequently the velocity distribution decreases. Fig 5 indicated the effects of magnetic parameter *M* on *f*′(*η*) for the blowing case. This Fig depicts the opposite results in blowing situation. Influence of squeezing parameter *S* on the velocity distribution for both cases of suction and blowing are shown in the Figs 8 and 9. It is analyzed that the velocity distribution *f*′(*η*) near the porous walls decreases and suction effects are dominant (see Fig 8). The flow enhances since upper wall is moving towards the stationary porous wall and pressure is produced. Therefore velocity distribution near the upper wall enhances in order to satisfy the mass conservation. Fig 9 indicated the effect of squeezing parameter *S* on the velocity distribution for case of blowing (*A* < 0). Fluid velocity is reduced due to the fact that lower wall acts as a retarding force in case of blowing (*A* < 0). On the other hand fluid velocity enhances in the upper half region of the channel. In fact in upper half channel the squeezing effects are dominant. Figs 10 and 11 display the influence of fluid parameter *α*_1_ and angle of inclination *ψ* on velocity distribution *f*′(*η*). It is noted that velocity distribution *f*′(*η*) increases near the porous wall when *η* ≤ 0.43 whereas it decreases when *η* > 0.43 for higher values of fluid parameter *α*_1_. In fact *α*_1_ is inversely proportional to the viscosity. For larger fluid parameter *α*_1_ the viscosity of fluid decreases and consequently the velocity distribution enhances. The behavior of angle of inclination *ψ* on the velocity profile *f*′(*η*) is illustrated in Fig 11. Here, *f*′(*η*) increases gradually when *ψ* enhances but there is a decrease in *f*′(*η*) when 0.4 ≤ *η* ≤ 1. It is due to the fact that by increasing angle of inclination *ψ* the influence of magnetic effects on fluid particle rises which enhances the Lorentz force. Therefore velocity distribution decreases. Also one can observed that *ψ* = 0 is the case when magnetic effect has no influence on the velocity distribution. For *ψ* = *π*/2 the fluid particles offered the maximum resistance.

### Temperature distribution

**Fig 12:**
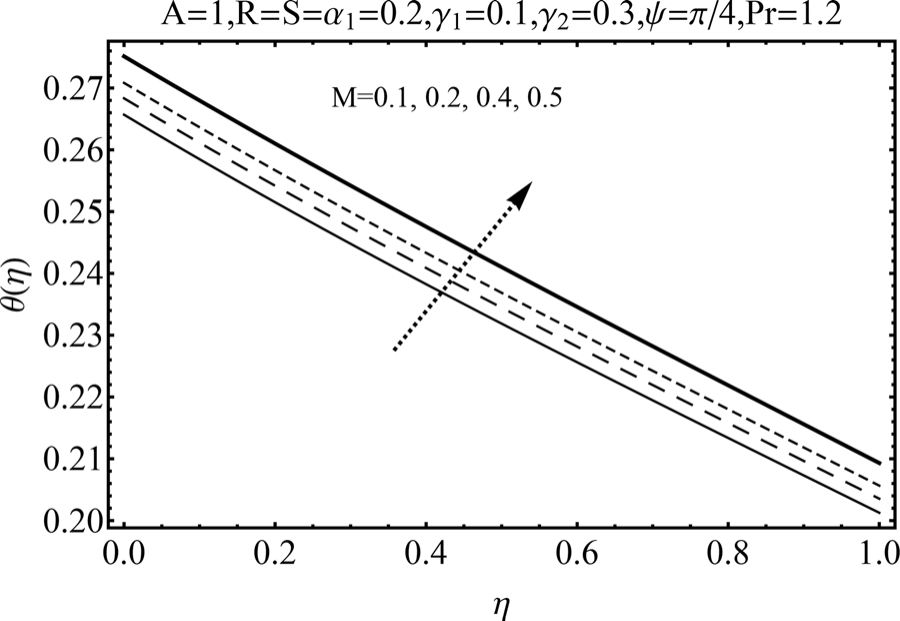
Influence of *M* on *θ*(*η*).

**Fig 13:**
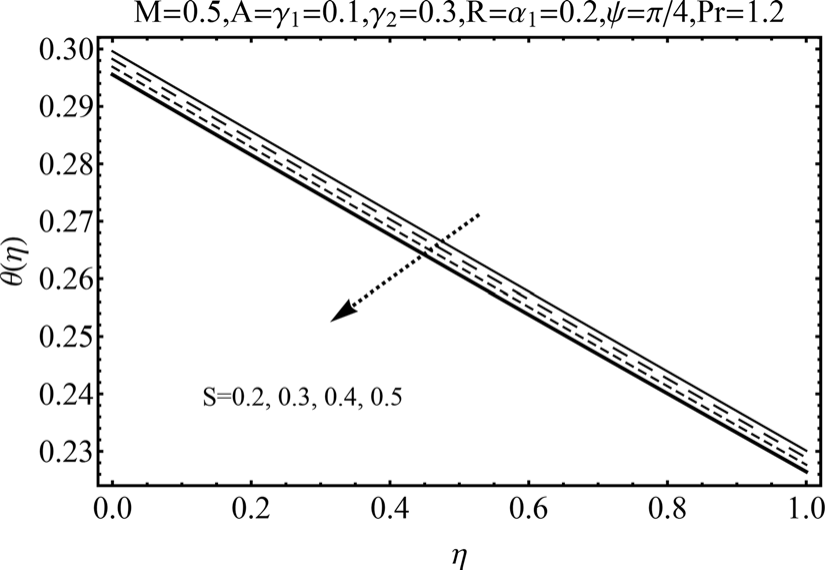
Influence of *S* on *θ*(*η*).

**Fig 14:**
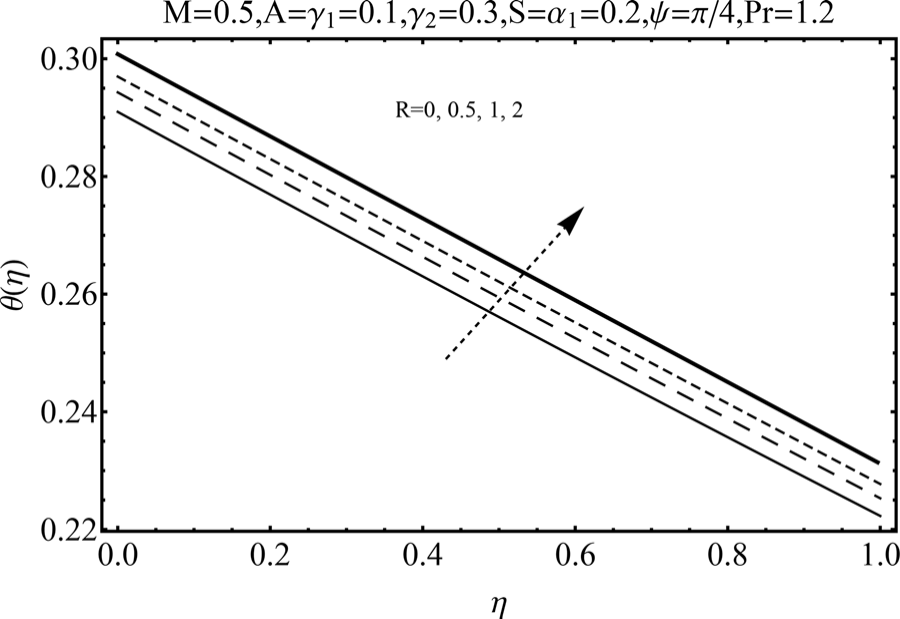
Influence of *R* on *θ*(*η*).

**Fig 15:**
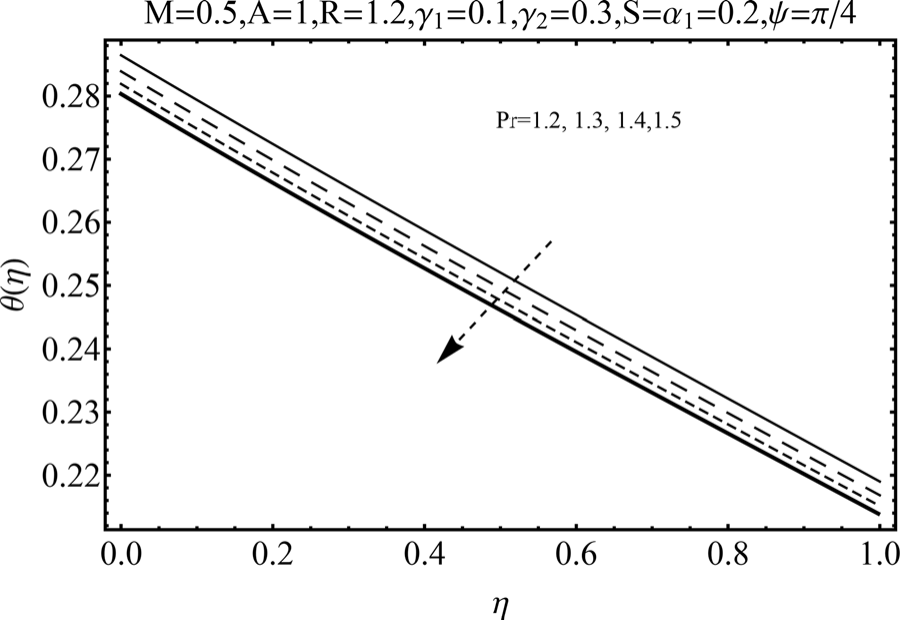
Influence of Pr on *θ*(*η*).

**Fig 16:**
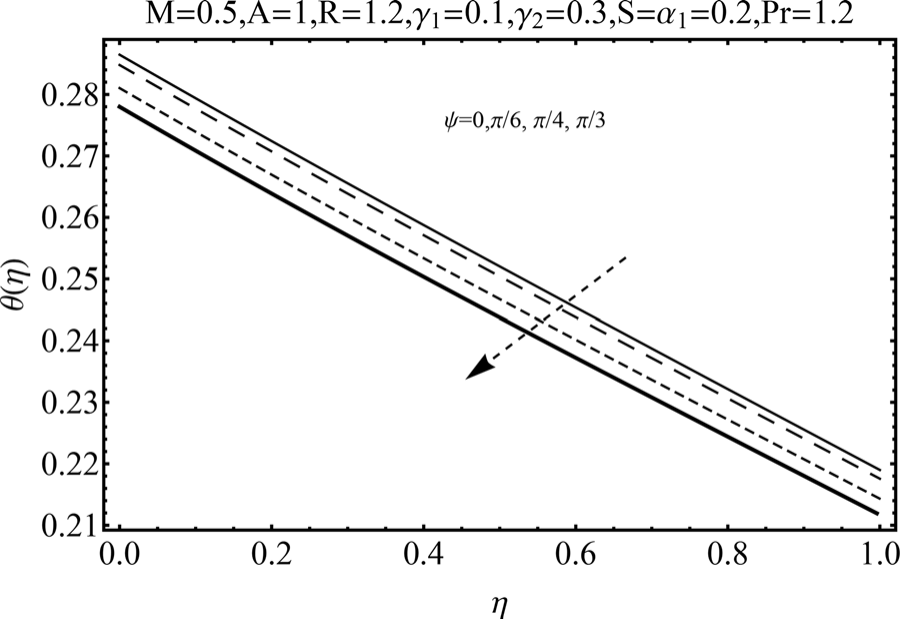
Influence of *ψ* on *θ*(*η*).

**Fig 17:**
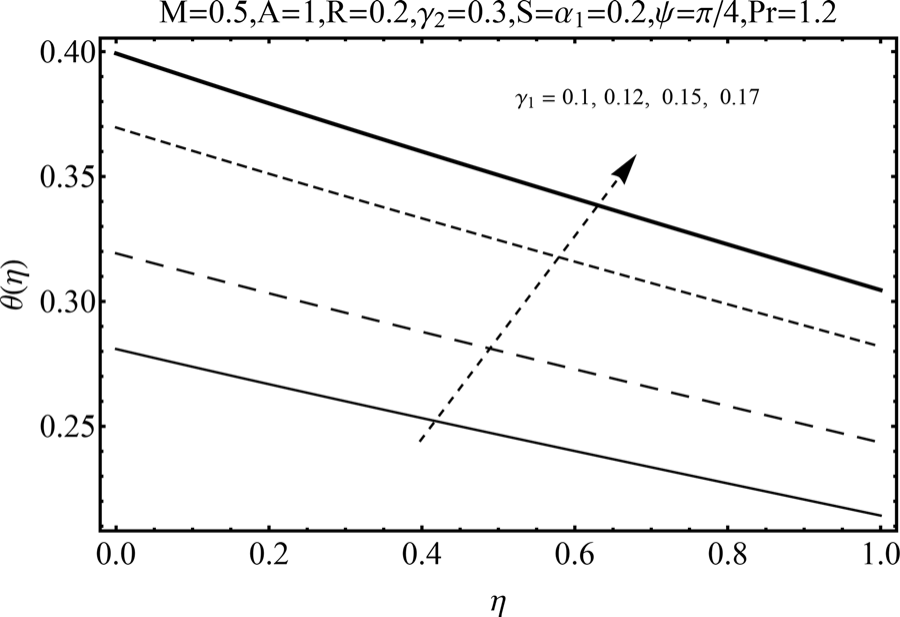
Influence of *γ*_1_ on *θ*(*η*).

**Fig 18:**
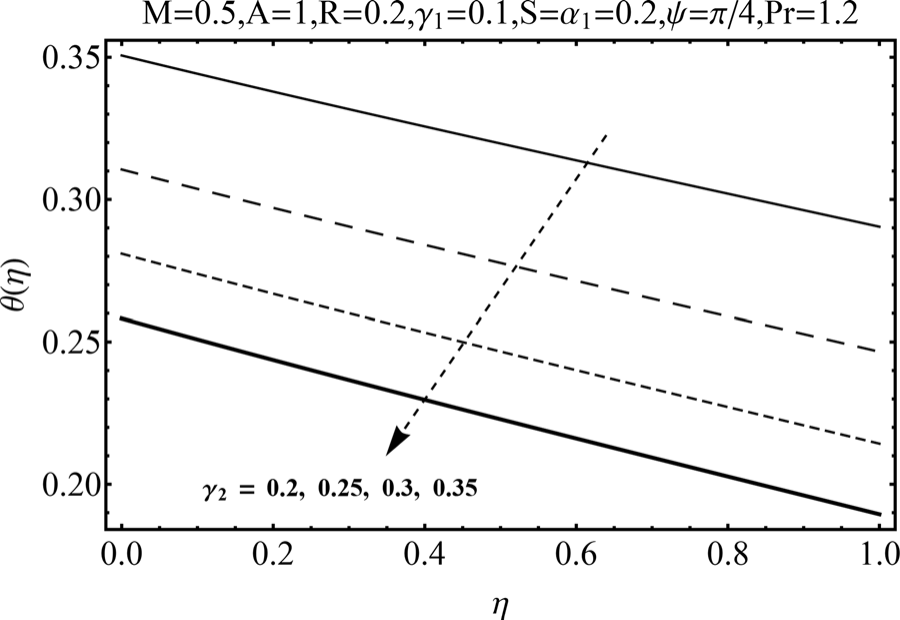
Influence of *γ*_2_ on *θ*(*η*).

Variation of magnetic parameter (*M*) on the temperature distribution *θ*(*η*) is shown in Fig 12. It is noted that temperature distribution increases for higher magnetic parameter *M*. This is due to the fact that an increase in *M* give rise to the resistive force (Lorentz force), and consequently the temperature distribution increases. Fig 13 illustrates the effect of squeezing parameter *S* on the temperature *θ*(*η*). This figure indicates that for larger values of squeezing parameter *S* the temperature distribution decreases. Higher values of squeezing parameter *S* indicate that kinematic viscosity decreases and depends upon the velocity and distance between disks. The effect of radiation parameter *R* on the temperature distribution *θ*(*η*) is visualized in Fig 14 by keeping other parameters fixed. Temperature distribution is an increasing function of radiation parameter *R*. The mean absorption coefficient decreases for higher thermal radiation parameter *R*. Therefore temperature distribution increases. Fig 15 is sketched to examine the influence of Prandtl number Pr on temperature profile. It is contemplated that an increase in Pr reduce the thermal boundary layer due to which the heat transfer rate enhances and as a result the temperature of fluid decreases. Fig 16 demonstrates the influence of angle of inclination (*ψ*) on temperature distribution *θ*(*η*). It is revealed that temperature profile *θ*(*η*) increases for higher values of inclination angle *ψ*. It is due to the fact that higher values of angle of inclination *ψ* corresponds to larger magnetic field which opposes the fluid motion. As a result the temperature profile *θ*(*η*) increases. Figs 17 and 18 display the impact of Biot number (*γ*_1_,*γ*_2_) on the temperature distribution *θ*(*η*). Fig 17 indicates the influence of Biot number (*γ*_1_) at the lower disk on temperature distribution. It is observed that larger *γ*_1_ leads to an increase in the temperature profile *θ*(*η*) and thermal boundary layer thickness. The Biot number is the ratio of the internal resistance of a solid to the thermal resistance of the disk surface. With the increase of the Biot number, the thermal resistance of the surface of disk decreases. Higher surface temperature is achieved due to increase in convection which makes the thermal effect to go deep in to the quiescent fluid. Fig 18 is plotted for various values of Biot number *γ*_2_ on temperature at the upper wall. It is noted that the fluid temperature decreases due to increasing convective heat loss at the upper disk.

### Skin Friction and local Nusselt number

Table 2 shows the comparison of numerical values of *f*″(1) with Domairry and Aziz [17] and Hayat et al. [5] for different values of squeezing parameter *S* and Hartman number *M*. It is observed that the present results are in excellent agreement with previous published data. Table 3 is prepared for the numerical values of skin friction coefficient for both upper and lower disks for various values of physical parameters. Magnitude of skin friction coefficient is increased for larger values of *S*, *M*, *α*_1_, and *ψ* at both upper and lower disks while it decreases for suction parameter *A* at both the upper and lower disks. The rate of heat transfer enhances for Pr, *R*, *S*, *γ*_1_, and *γ*_2_ at the lower disk. On the upper disk the rate of heat transfer decreases by increasing the values of Pr and *S* while it increases for larger values of *R*, *γ*_1_, and *γ*_2_. Table 4 is drawn for the numerical values of local Nusselt number for the different physical parameters.

**Table 2:** Comparison between HAM and HPM in limiting situations for different values of squeezing parameter *S* and Hartman number *M* when other parameters are fixed.

S	M	*f*″(1)		
		Hayat et al. [5]	Domairry and Aziz [8]	present work
0.1	1	3.02725	3.02725	3.02725
0.2		3.00560	3.00560	3.00560
0.3		2.98468	2.98468	2.98468
0.4		2.96449	2.96449	2.96449
0.1	0	2.97682	2.97682	2.97682
	1	3.02725	3.02725	3.02725
	2	3.17424	3.17424	3.17424
	3	3.40620	3.40620	3.40620

**Table 3:** Numerical data of skin friction coefficient at upper and lower disks for different values of parameters.

*A*	*S*	*M*	*α*_1_	*ψ*	$\frac{H^{2}}{r^{2}}Re_{r}C_{fr0}$	$-\frac{H^{2}}{r^{2}}Re_{r}C_{fr1}$
0.1	0.3	5	0.1	*π* / 3	3.5198	3.4281
0.2					2.7138	2.5778
0.3					1.8575	1.7229
0.1	0	5	0.1	*π* / 3	3.4803	3.4087
	0.2				3.5066	3.4217
	0.4				3.5329	3.4346
0.1	0.3	2	0.1	*π* / 3	2.9686	2.8953
		3			3.1070	3.0302
		4			3.2928	3.2101
0.1	0.3	5	0.05	*π* / 3	3.3130	3.2565
			0.15		3.7260	3.6008
			0.16		3.7672	3.6355
0.1	0.3	5	0.1	*π* / 6	3.0888	3.0125
				*π* / 4	3.3101	3.2268
				*π* / 2	3.7193	3.6184

**Table 4:** Numerical values of Nusselt number at both upper and lower disks for different values of parameters.

Pr	*R*	*S*	*γ*_1_	*γ*_2_	${\left(1-at\right)}^{\frac{1}{2}}Nu_{r0}$	${\left(1-at\right)}^{\frac{1}{2}}Nu_{r1}$
1	0.2	0.3	0.2	0.1	0.080403	0.078528
1.2					0.080652	0.078399
1.3					0.080776	0.078335
1	0.3	0.3	0.2	0.1	0.088736	0.086861
	0.4				0.097069	0.095195
	0.5				0.105400	0.103530
1	0.2	0.4	0.2	0.1	0.080818	0.078313
		0.5			0.081235	0.078098
		0.6			0.081654	0.077882
1	0.2	0.3	0.7	0.1	0.103974	0.101549
			0.8		0.105521	0.103059
			0.9		0.106756	0.104266
1	0.2	0.3	0.2	0.3	0.137044	0.133847
				0.4	0.150277	0.146771
				0.6	0.166338	0.162458

## Conclusions

Impact of thermal radiation in the squeezing flow of second grade fluid with convective boundary condition is explored. The following points of presented analysis are worthmentioning.

- Velocity profile decreases while the temperature distribution increases for larger magnetic parameter.
- Effects of angle of inclination, Prandtl number and magnetic parameter are qualitatively similar.
- Effect of Prandtl number on temperature field is opposite to that of thermal radiation parameter *R*.
- Temperature distribution enhances for larger radiation parameter *R*.
- Effects of magnetic and squeezing parameters on velocity distribution having similar behavior for both suction and blowing cases.
- Skin friction coefficient increases for higher values of *S*, *M*, *α*_1_, and *ψ*.
- For the larger values of *R*, *γ*_1_, and *γ*_2_ the magnitude of local Nusselt number increases at both upper and lower disks.
